# Teaching medical residents to collaborate with specialist consultants

**DOI:** 10.5116/ijme.5d6b.85bc

**Published:** 2019-09-20

**Authors:** Orimisan S. Adekolujo, Steven E. Roskos, Oyebimpe O. Adekolujo

**Affiliations:** 1 Department of Medicine, McLaren Flint, 401 S Ballenger Hwy, Flint, MI, USA; 2 Department of Family Medicine, College of Human Medicine, Michigan State University, East Lasing, MI, USA

**Keywords:** Teaching, medical residents, collaborate, specialist consultants

## Introduction

An 83 -year-old male patient with a past history of essential hypertension, diabetes mellitus type 2 and subdural hemorrhage complicating a fall was admitted because of acute congestive heart failure. Electrocardiography revealed atrial fibrillation with rapid ventricular response. Echocardiography showed left ventricular ejection fraction of 50%. He was commenced on diuresis with intravenous furosemide and rate control of the atrial fibrillation was achieved with intravenous diltiazem. He improved significantly and oral medications including furosemide, metoprolol, and lisinopril were commenced in preparation for discharge. The cardiologist recommended anticoagulation with warfarin given the patient’s CHADS score of 4 which put him at high risk for thromboembolism. The medical resident in charge of the patient was concerned about the use of warfarin in this patient given patient’s previous history of fall and the resultant intracranial bleed. He thought it may not be safe to start anticoagulation in this patient.  What should he do?

We become uncomfortable whenever we ask a trainee for the rationale for his decision on patient care and get the reply: “It was the consultant’s recommendation”. The ability to collaborate with consultants is a necessary skill for all physicians and accreditation bodies for graduate medical education in many countries require medical residents to acquire the skills necessary to interact and collaborate with specialists during a consultation in patient care.^1^^,^^2^  However, we have observed that many medical residents struggle in their interactions with consultants and often accept recommendations without probing the underlying rationale, resulting in lost learning opportunities and suboptimal patient care.

The 5Cs of Consultation (Contact, Communication, Core Question, Collaboration and Closing the Loop) has been validated as an effective tool for training medical students and residents in the skills necessary to effectively interact with consultants.^3^^,^^4^^,^^5^ We have found that medical residents struggle most with the fourth C, Collaboration: “Planning a course of action that results from the discussion between the consulting physician and the consultant, including any alteration of management or testing”.^5^ Overwhelmingly, trainees tend to accept consultants’  recommendations as sacred truth, something to be believed and not to be questioned. Residents may find it difficult to question recommendations from consultants in part because of the hierarchical nature of medicine, especially in a training environment. Attending physicians of all specialties, and some specialists, in particular, are revered and feared by medical residents. This fear may be unfounded or may be based on past experience where a resident felt inadequate because he/she was found lacking in some knowledge or skill by the attending physician or specialist.^6^ Because of this fear, residents may fail to ask necessary questions to clarify a recommendation or understand the rationale behind it. There are good reasons to seek to understand the rationale for a consultant’s recommendation. First, every consultation is a potential learning experience, which can be facilitated if a medical resident asks questions to understand the clinical reasoning behind a recommendation. Second, a consultant’s recommendation may be inappropriate for the clinical situation or inconsistent with the patient’s expressed values.^7^ A consultant may be limited by a narrow range of expertise or experience or may have limited knowledge of a particular patient including the patient’s medical history, values and preferences compared with the primary physician on the case.^7^

Teaching physicians are responsible for helping residents develop the skills necessary to interact with consultants and become adept at considering the applicability of the consultants’ recommendations to an individual patient. To fulfill the aforementioned role, medical educators must demonstrate the critical role of teamwork in safe and effective patient care by fostering an atmosphere of openness and trust among all caregivers, with individual and mutual accountability.^6^ We should encourage open discussion of issues relating to patient care especially in areas of uncertainty. We must recognize our responsibility to explain the rationale for our patient care decisions and clearly point out areas of uncertainty, especially when asked by a trainee. The team should only make a plan after consideration of input from every member.  Trainees are more likely to engage consultants with the purpose of clarifying recommendations after experiencing and witnessing such interactions with their teachers.

The complexity of the healthcare system mandates that the primary physician who knows the patient best play an advocate role. We should teach residents to take up this role by example; speaking on behalf of our patients’ best interests in exploring management options. Playing an advocate role is especially important when multiple specialists are involved in the care of a patient. Attending physicians should use such instances to demonstrate collaborative interactions with specialists. For instance, a phone or face-to-face conversation during a ward round demonstrating how a primary physician’s input can be crucial in helping a specialist shape his recommendation will go a long way to help medical residents overcome the prohibitive power dynamics between specialties.

In this kind of collegial environment, every recommendation from a consultant should be evaluated for its relevance to the particular clinical situation and patient. A framework for such evaluation includes the bioethical principles of non-maleficence, beneficence, and respect for the patient’s autonomy.^8^  Any recommended intervention should have a low likelihood of causing harm to the patient and a high probability of delivering promised benefit, given the totality of the patient’s medical history.  Of course, the patients’ rights to make decisions regarding their healthcare must always be respected.  In addition, recommendations should be judged by their consistency with the patient’s values and preferences, and their actionability.

In the case presented above, the on-call resident had difficulty in deciding the best course of action. Thus, he reached out to one of us for advice. We reviewed the patient’s medical history and had a discussion on care preferences with the patient and his family. Relevant aspects of the patient’s medical history including recurrent falls and previous subdural hemorrhage were verified. The patient mentioned he fell three times in the month before admission and family were worried about the possibility of another intracranial bleed while on anticoagulation.  Given that the heightened risk of bleeding and resultant harm was greater in comparison to the absolute risk of thromboembolism and the likely benefit from anticoagulation, and in line with patient preference, we decided not to start warfarin and prescribed low dose aspirin instead. We communicated this decision and rationale to the cardiologist, who agreed with the new plan. This case demonstrated effective collaboration and adherence to the above ethical principles in the evaluation of a consultant’s recommendation.

In conclusion, by role modeling teamwork in clinical decision-making with the application of common bioethical principles, medical educators can facilitate the development of skills that are critical for trainees to collaborate effectively with consultants and to weigh the appropriateness of consultants’ recommendations in the care of their patients.

### Acknowledgments

The authors thank Radhika Kakarala, MD and Susan Smith, MD for their suggestions on this article.

### Conflict of Interests

The authors declare that they have no conflict of interests.
